# Inhibition of Ocular Aldose Reductase by a New Benzofuroxane Derivative Ameliorates Rat Endotoxic Uveitis

**DOI:** 10.1155/2014/857958

**Published:** 2014-11-11

**Authors:** C. Di Filippo, M. V. Zippo, R. Maisto, M. C. Trotta, D. Siniscalco, B. Ferraro, F. Ferraraccio, C. La Motta, S. Sartini, S. Cosconati, E. Novellino, C. Gesualdo, F. Simonelli, S. Rossi, M. D'Amico

**Affiliations:** ^1^Department of Experimental Medicine, Section of Pharmacology “L. Donatelli”, Second University of Naples, Via Costantinopoli 16, 80138 Naples, Italy; ^2^Department of Clinical, Public and Preventive Medicine, Second University of Naples, Via Armanni 5, 80138 Naples, Italy; ^3^Department of Pharmacy, University of Pisa, Via Bonanno 6, 56126 Pisa, Italy; ^4^DiSTABiF, Second University of Naples, Via Vivaldi 43, 81100 Caserta, Italy; ^5^Department of Pharmacy, University of Naples “Federico II”, Via Montesano 49, 80131 Naples, Italy; ^6^Multidisciplinary Department of Medical, Surgical and Dental Specialities, Second University of Naples, Via Pansini 5, 80131 Naples, Italy

## Abstract

The study investigated the effects of the aldose reductase (AR) inhibitor benzofuroxane derivative 5(6)-(benzo[*d*]thiazol-2-ylmethoxy) benzofuroxane (herein referred to as BF-5m) on the biochemical and tissue alterations induced by endotoxic uveitis in rats. BF-5m has been administered directly into the vitreous, in order to assess the expression and levels of (i) inflammatory markers such as the ocular ubiquitin-proteasome system, NF-*κ*B, TNF-*α*, and MCP-1; (ii) prooxidant and antioxidant markers such as nitrotyrosine, manganese superoxide dismutase (MnSOD), and glutathione peroxidase (GPX); (iii) apoptotic/antiapoptotic factors caspases and Bcl-xl; (iv) markers of endothelial progenitor cells (EPCs) recruitment such as CD34 and CD117. 5 *μ*L of BF-5m (0.01; 0.05; and 0.1 *μ*M) into the right eye decreased in a dose-dependent manner the LPS-induced inflammation of the eye, reporting a clinical score 1. It reduced the ocular levels of ubiquitin, 20S and 26S proteasome subunits, NF-*κ*B subunits, TNF-*α*, MCP-1, and nitrotyrosine. BF-5m ameliorated LPS-induced decrease in levels of MnSOD and GPX. Antiapoptotic effects were seen from BF-5m by monitoring the expression of Bcl-xl, an antiapoptotic protein. Similarly, BF-5m increased recruitment of the EPCs within the eye, as evidenced by CD34 and CD117 antibodies.

## 1. Introduction

Uveitis, the inflammation of the uvea in the eye, is among the leading causes of blindness and vision loss in the world [1–4]. This disease affects people of all ages, gender, and race, though sex preponderance can be observed in some cases [3, 4]. It also affects the working population between 20 and 59 years [5, 6]. Because of the variability of causes, the etiology of the uveitis is difficult to define. This pathology is characterized by elevated levels of proinflammatory cytokines in ocular tissue, which involve the production of inflammatory molecules and reactive oxygen species (ROS) [7]. The experimental animal model closest to human uveitis is the acute form of bacterial endotoxin, lipopolysaccharide- (LPS-) induced uveitis (EIU) [3, 4, 8]. LPS increases the systemic expression of several inflammatory molecules such as TNF-*α*, IL-6, MIF, IFN-*γ*, and MCP-1 and the production of nitric oxide. High levels of these mediators induce, after the rupture of the blood-ocular barrier, the infiltration of leukocytes and monocytes in ocular tissues promoting the development of EIU [9].

The literature reports that the inhibition of aldose reductase (AR), a key enzyme of the polyol pathway able to convert the excess glucose into sorbitol with the consequent formation of ROS, prevents or decreases the acute form of the ocular inflammation induced by LPS in rats, through decreased oxidative stress signals [10]. Various aldose reductase inhibitors (ARIs) have been tested against uveitis [3, 4, 7, 11–13]. We recently identified a novel class of nonhydantoin noncarboxylic acid inhibitors, featuring the benzofuroxane core [14, 15] as new scaffold interacting with the so-called AR anion site. Merging submicromolar AR inhibitory activities with significant ROS scavenging properties, these compounds could represent the ideal therapeutic treatment for EIU being able to prevent oxidative stress-induced inflammatory events.

For these reasons, and having in mind the presence of the blood-ocular barrier which may limit the achievement of therapeutic concentrations of these inhibitors within the vitreous and chorioretina after systemic administration, we tested the most effective compound of the whole series, namely, 5(6)-(benzo[*d*]thiazol-2-ylmethoxy)benzofuroxane (herein referred to as BF-5m), injected directly into the vitreous in order to assess whether the inhibition of the local AR ameliorates the biochemical and tissue alterations induced by EIU. We investigated the effects that the compound may have on the ocular levels of (i) inflammatory markers such as the ocular ubiquitin-proteasome system and NF-*κ*B pathway; (ii) prooxidant and antioxidant markers such as nitrotyrosine, MnSOD, and GPX; (iii) proapoptotic/antiapoptotic factors caspases and Bcl-xl protein; (iv) markers of endothelial progenitor cells (EPCs) recruitment such as CD34 and CD117.

## 2. Materials and Methods

### 2.1. Drug

**Figure 1:**
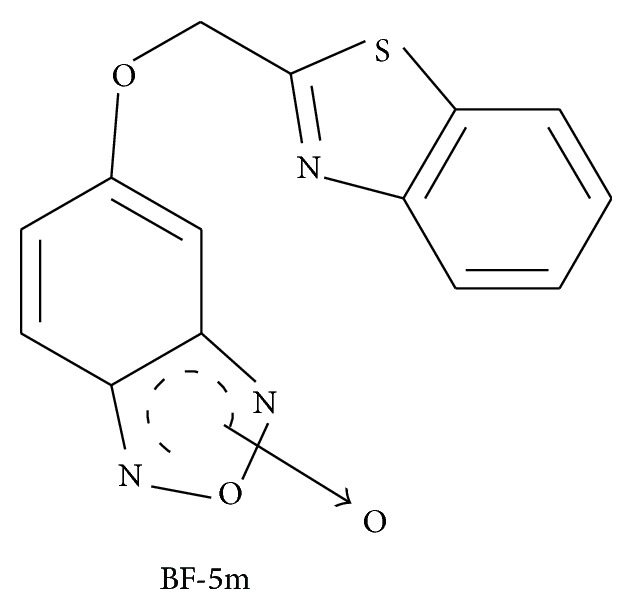
Chemical structure of the 5(6)-(Benzo[*d*]thiazol-2-ylmethoxy)benzofuroxane derivative BF-5m. Aldose reductase IC_50_ = 0.42 ± 0.01 *μ*M in vitro.

BF-5m, 5(6)-(benzo[*d*]thiazol-2-ylmethoxy)benzofuroxane (Figure 1), was synthesized at the Department of Pharmacy of the University of Pisa, Italy, following a previously reported procedure [15]. Briefly, alkylation of the commercially available 4-amino-3-nitrophenol with chloroacetonitrile, in the presence of anhydrous potassium carbonate, provided the 2-(4-amino-3-nitrophenoxy) acetonitrile which, through reaction with o-aminothiophenol, gave the key intermediate 4-[(benzo[*d*]thiazol-2-yl)methoxy]-2-nitrobenzenamine. Treatment with sodium nitrite in concentrated hydrochloric acid, and then with sodium azide in water, gave the corresponding azido-derivative, which cyclized to the target inhibitor, 5(6)-(benzo[*d*]thiazol-2-ylmethoxy) benzofuroxane, when heated under reflux in an acetic acid solution.

### 2.2. Induction of EIU

Male Sprague-Dawley rats (Harlan, Italy) (420–450 gr) were injected subcutaneously into the footpad with 200 *μ*g of lipopolysaccharide (LPS,* Salmonella minnesota*, Sigma St Louis, MO, USA) in 0.1 mL of sterile pyrogen-free saline for induction of EIU. 1 h after LPS injection, under pentobarbital anaesthesia (45 mg/kg i.p. in saline), the pupils were dilated by instillation of one drop of tropicamide 5%; then local anaesthesia was induced with one drop of tetracaine 1% followed by intravitreal injection of the tested compound in the right eye. This was executed by using sterile syringes fitted with a 30-gauge needle (Micro-fine, BD, Italy) and 5 *μ*L [16] of three different BF-5m concentrations, 0.01, 0.05, and 0.1 *μ*M, in the range used in previous studies [15]. The compound was reconstituted in 1% dimethyl sulfoxide (DMSO, Sigma, Italy) 2 h prior to LPS injection and injected into the vitreous 1 h after LPS. The following 6 experimental groups were considered (*n* = 6 for each group): vehicle (saline); vehicle (1% DMSO); LPS+saline; LPS+BF-5m (0.01; 0.05; 0.1 *μ*M/rat). Rats were killed 24 h after treatment and evaluated for clinical signs and biochemical markers. All the procedures were approved by the local Ethic Committee, number 2108/12.

### 2.3. Clinical Score Attributed to EIU

The development of uveitis was determined 24 h after the administration of the compound following the method reported by Rossi et al. [17]. The clinical signs of uveitis were scored from 0 to 4, and uveitis was considered positive when the score assigned was >1.

### 2.4. Eye Samples

Harvested eyes were cut in two halves: one half was immediately frozen in liquid nitrogen for the biochemical assays, and the other half was immediately fixed by immersion in 10% buffered formalin and paraffin-embedded for immunohistochemistry, as previously described [17].

### 2.5. Western Blotting Assay

Western blot technique was performed in agreement with our previous work [17]. The primary antibodies used were antiproteasome subunits (Fl-76, anti-20S, and anti-26S), anti-NF-*κ*B p65 (C-20), anti-NF-*κ*B p50 (H-119) and p105 (H-119), anti-glutathione peroxidase (GPX), anti-manganese-superoxide dismutase (Mn-SOD), and anti-Bcl-xl. These primary antibodies were purchased from Santa Cruz Biotec (USA). Secondary antibodies were conjugated with HRP horseradish peroxidase, donkey polyclonal to rabbit IgG, goat anti-mouse, and goat anti-rabbit, and were all purchased from Santa Cruz Biotec (USA). Results are expressed in densitometric units.

### 2.6. ELISA Assay

A commercial colorimetric ELISA kit (R&D systems, USA) was used to quantitatively assay the levels of ocular tumor necrosis alpha (TNF-*α*) and monocyte chemoattractant protein-1 (MCP-1) according to the manufacturer's instructions. 200 *μ*L of eye homogenate was used. Briefly, frozen tissues were homogenized in PBS with protease inhibitors, added of Triton X-100 to a final concentration of 1% and centrifuged for 5 min at 10,000 ×g at 4°C.

### 2.7. Immunohistochemistry

Antinitrotyrosine antibodies were used for evaluating oxidative stress and CD117 and CD34 antibodies for recruitment of EPCs (Santa Cruz Biotec, USA). The ocular tissues were incubated with specific primary antibodies, washed in PBS, and incubated with secondary antibody. The ocular samples were analyzed by an expert pathologist (variability 6%); every sample was visualized at 200x magnification. The number of CD34^+^, CD117^+^, and nitrotyrosine positive particles per area was analyzed in 20 microscopic fields under 200x.

### 2.8. RNA Extraction and RT-PCR

Total RNA was extracted from homogenized eye using an RNA Tri-Reagent (Molecular Research Center Inc., Cincinnati, OH) according to the manufacturer's protocol. The extracted RNA was subjected to* DNase* I treatment at 37°C for 30 min. The total RNA concentration was determined by UV spectrophotometer. The mRNA levels were measured by RT-PCR amplification, as previously reported [18]. RT minus controls were carried out to check potential genomic DNA contamination. These RT minus controls were performed without using the reverse transcriptase enzyme in the reaction mix. Sequences for the rat mRNAs from GeneBank (DNASTAR INC., Madison, WI) were used to design primer pairs for RT-PCRs (OLIGO 4.05 software, National Biosciences Inc., Plymouth, MN). Each RT-PCR was repeated at least three times to achieve best reproducibility data. The measured mRNA levels were normalised with respect to hypoxanthine-guanine phosphoribosyl transferase (HPRT), chosen as housekeeping gene. The HPRT gene expression did not change in several experimental conditions [18]. To our knowledge there is no molecular evidence for variation in HPRT mRNA-levels in this model. The gene expression values were expressed as arbitrary units ± SE. Amplification of genes of interest and HPRT was performed simultaneously. PCR products were resolved into 2.0% agarose gel. A semiquantitative analysis of mRNA levels was carried out by the Gel Doc EZ UV System (Bio-Rad, Hercules, CA). Total RNA was extracted from the eye of vehicle-, LPS- and BF-5m-treated rats and reverse transcribed into cDNA using Superscript reverse transcriptase system. The expression of caspase 3 and caspase 8 was quantified by qPCR using commercially available rat primers. HPRT was used as internal control. Results are expressed as arbitrary units based on calculation of 2^−ΔΔCt^ method. Relative amount of target genes were normalized to HPRT and to vehicle.

### 2.9. Statistical Analysis

Data are expressed as means ± standard error of the mean (SEM). Student's *t*-test (when only two groups were compared) or one-way ANOVA followed by Dunnett's test (more than two experimental groups) was used. *P* < 0.05 was considered statistically significant.

## 3. Results

### 3.1. Effects of BF-5m on EIU Clinical Score

**Figure 2:**
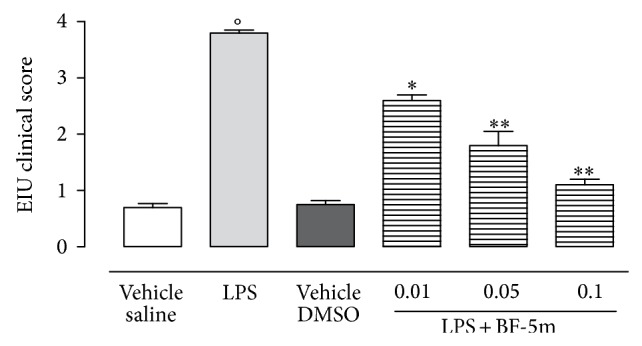
Clinical score attributed to EIU after intravitreal BF-5m. Sprague-Dawley rats were treated with vehicle (saline), LPS (200 *μ*g/rat), vehicle (1% DMSO), and LPS+BF-5m (0.01; 0.05; 0.1 *μ*M/rat) 1 h following LPS treatment and were evaluated 24 h after injections. Clinical score to EIU has been reported in the test (see Material and Methods). Values are mean ± SEM, of *n* = 6 observation for each experimental group. ^*^ *P* < 0.05 and ^**^ *P* < 0.01 compared with LPS-treated group; °*P* < 0.01 versus vehicle saline group.

LPS-injected rats showed alterations of vascular intermediate membrane of the eye with a clinical score of 4 (Figure 2). BF-5m injected into the vitreous at three different concentrations (0.01; 0.05; and 0.1 *μ*M) decreased the LPS-induced inflammation of this eye in a dose-dependent manner, reporting a clinical score 1.1 (Figure 2).

### 3.2. BF-5m and EIU Inflammatory Markers

**Figure 3:**
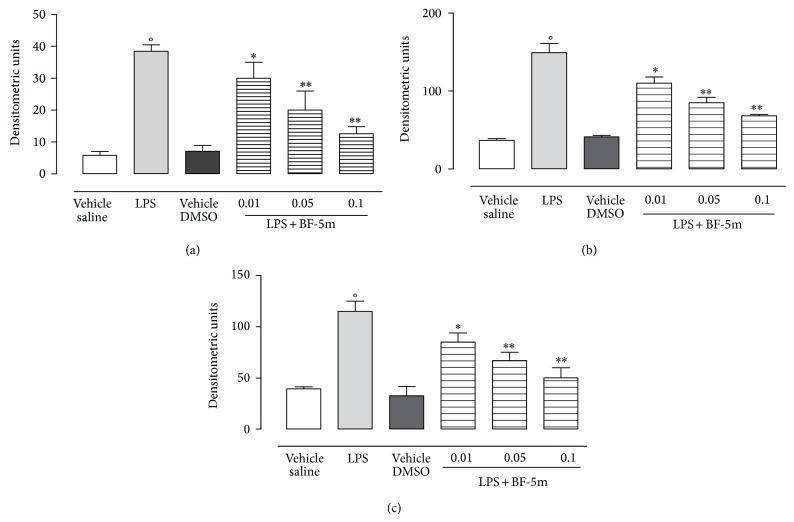
Western blotting analysis for ubiquitin, proteasome 20S, and proteasome 26S subunits. Injection of BF-5m (0.01; 0.05; 0.1 *μ*M/rat) into the vitreous of LPS-treated rats dose-dependently reduced the expression of ubiquitin (a), protesome 20S (b), and proteasome 26S (c). Results are expressed as densitometric units and represented the mean ± SEM of *n* = 6 observations for each group. ^*^ *P* < 0.05 and ^**^ *P* < 0.01 versus LPS-treated rats; °*P* < 0.01 versus vehicle saline group.

**Figure 4:**
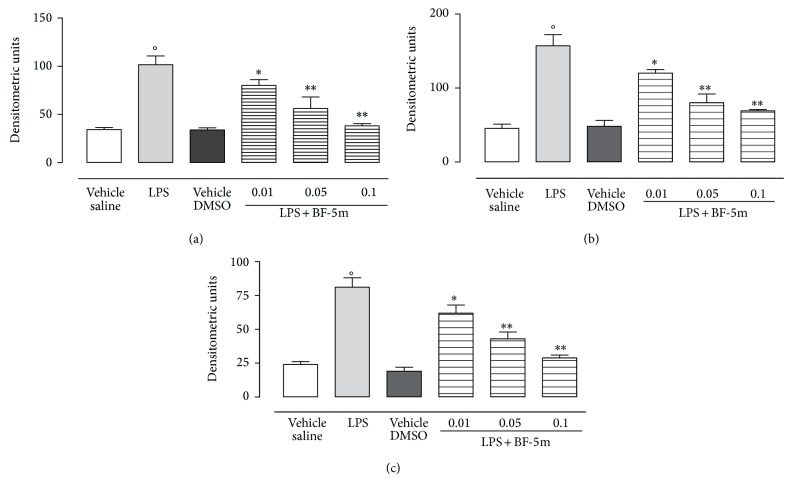
Western blotting analysis for NF-*κ*B. Injection of BF-5m (0.01; 0.05; 0.1 *μ*M/rat) into the vitreous 24 h after LPS-treatment of the rats reduced the expression of activated NF-*κ*B 105 (a), p50 (b), and p65 (c). Results are expressed as densitometric units and represented the mean ± SEM of *n* = 6 observations for each group. °*P* < 0.01 versus vehicle saline group ^*^ *P* < 0.05 and ^**^ *P* < 0.01 versus LPS-treated rats.

Western blotting analysis revealed the highest expression of markers of inflammation such as ubiquitin, 20S and 26S proteasome subunits, and NF-*κ*B subunits in the eyes following LPS treatment. These markers were significantly and dose-dependently reduced by BF-5m. The tested compound expressed maximal action at the concentration of 0.1 *μ*M (Figures 3 and 4). 0.01 and 0.05 *μ*M BF-5m had less effects on proteasome and NF-*κ*B (Figures 3 and 4).

**Figure 5:**
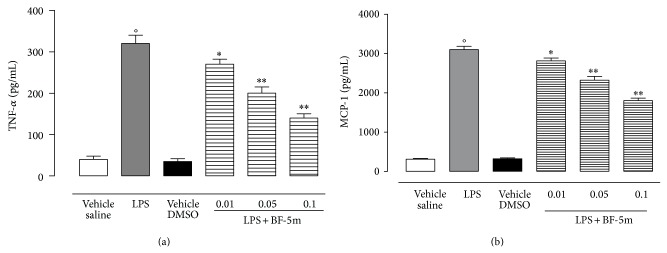
Ocular levels of tumor necrosis factor alpha (TNF-*α*) and monocyte chemoattractant protein-1 (MCP-1) within the tissue of rats with EIU, evaluated 24 h after the administration of vehicle (saline), vehicle (DMSO), LPS (200 *μ*g/s.c.), or LPS+BF-5m (0.01; 0.05; 0.1 *μ*M/rat) into the vitreous. The values are expressed as mean ± SEM of *n* = 6 observations for each group. ^*^ *P* < 0.05 and ^**^ *P* < 0.01 versus LPS-treated rats; °*P* < 0.01 versus vehicle saline group.

In addition, ELISA showed a dose-dependent reduction of the ocular levels of the cytokine TNF-*α* and of the chemokine MCP-1 following intravitreal BF-5m compared with the levels expressed into the ocular tissue of LPS-treated animals (Figure 5).

### 3.3. BF-5m Affects the Oxidative Stress Induced by EIU

**Figure 6:**
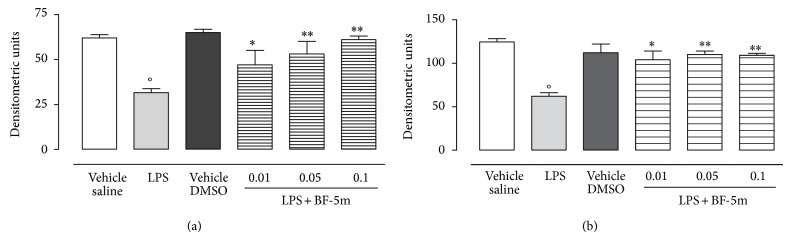
Western blotting analysis for Mn-SOD and GPX. Expression of manganese superoxide dismutase (Mn-SOD) (a) and glutathione peroxidase (GPX) (b) into the vitreous of rats 24 h after treatment with LPS or LPS+BF-5m (0.01; 0.05; 0.1 *μ*M/rat) compound. Densitometric units as calculated from WB traces are expressed as mean ± SEM of *n* = 6 observations for each group. ^*^ *P* < 0.05 and ^**^ *P* < 0.01 versus LPS-treated rats; °*P* < 0.01 versus vehicle saline group.

Investigation on eye tissue homogenates of rats with EIU showed significantly decreased levels of MnSOD (−44%) and GPX (−40%) induced by LPS with respect to the vehicle (saline) group (Figure 6). These decreases were almost completely abolished by intravitreal injection of BF-5m, with a maximum effect on MnSOD (42%) and GPX (36%) expression at 0.1 *μ*M concentration (Figure 6).

**Figure 7:**
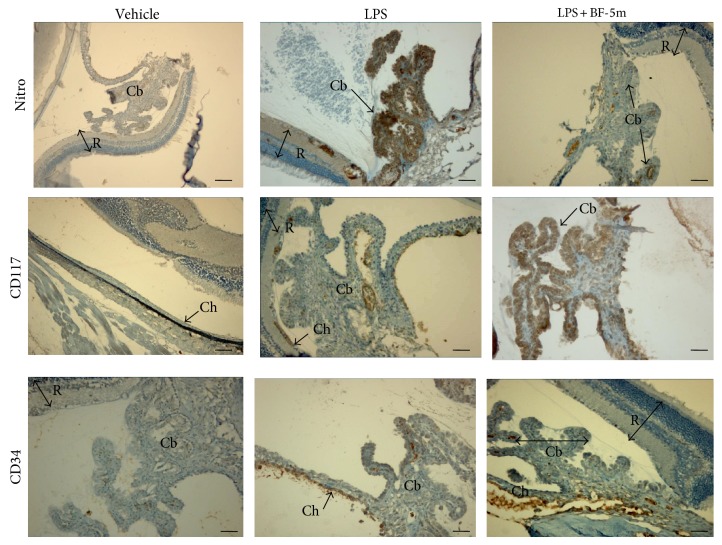
Representative immunohistochemistry for nitrotyrosine, CD117-ckit, and CD34 in the eye structures of rats treated with vehicle, LPS, and BF-5m (0.05 *μ*M/rat). Intravitreal BF-5m increases CD34^+^ and CD117^+^ immunostaining into the eye, while reducing the nitrotyrosine. Nitro: nitrotyrosine; R: retina; Ch: choroid; Cb: ciliary bodies. Scale bar, 50 *μ*m. 200x magnification.

**Figure 8:**
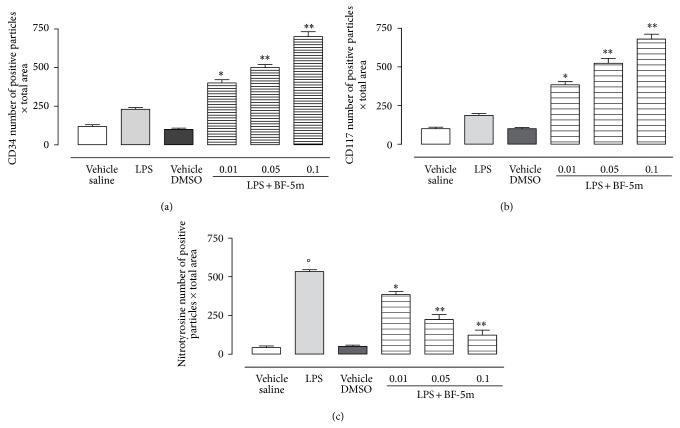
Number of CD34^+^, CD117^+^, and nitrotyrosine positive particles per area analyzed by immunohistochemistry. 20 microscopic fields were counted under 200x magnification in vehicle, LPS, and LPS+BF-5m 0.01; 0.05; 0.1 *μ*M/rat. Values are mean ± SEM of *n* = 6 observations for each group. ^*^ *P* < 0.05 and ^**^ *P* < 0.01 versus LPS-treated group; °*P* < 0.01 versus vehicle saline group.

To define oxidative damage within the eyes of EIU rats treated with BF-5m, immunohistochemistry was used to evaluate the nitrotyrosine expression 24 h after LPS injection, time required for developing the acute phase of inflammation. The expression of nitrotyrosine that was mainly localized into the ciliary bodies and choroid augmented significantly in the eyes of LPS group (Figures 7 and 8). Treatment with BF-5m significantly reduced the expression of this marker with respect to vehicle alone (Figures 7 and 8). Interestingly, this reduction induced by BF-5m was paralleled by increased expression of CD34 and CD117, markers of EPCs recruitment into the eye (Figures 7 and 8).

### 3.4. Antiapoptotic Effects of BF-5m

**Figure 9:**
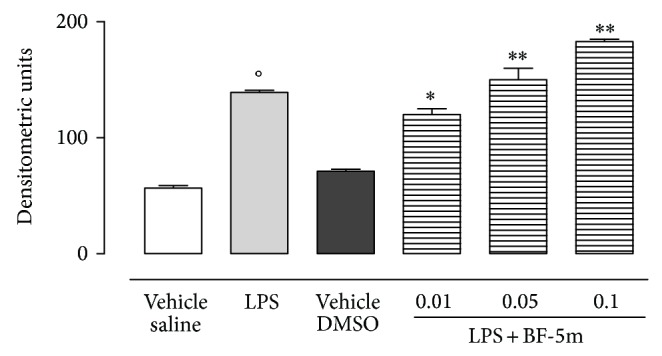
Western blotting for Bcl-xl. Expression of the antiapoptotic protein Bcl-xl in eye tissue of rats treated with LPS or LPS+BF-5m (0.01; 0.05; 0.1 *μ*M/rat). Values are mean ± SEM of *n* = 6 observations for each group. ^*^ *P* < 0.05 and ^**^ *P* < 0.01 versus LPS-treated rats; °*P* < 0.01 versus vehicle (saline) group.

**Figure 10:**
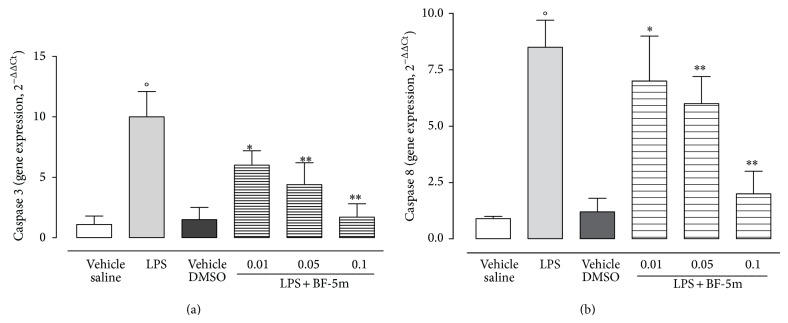
RT-PCR analysis for caspase 3 and caspase 8. Caspase 3 and caspase 8 in ocular tissue following treatment with BF-5m (0.01; 0.05; 0.1 *μ*M/rat). Total RNA was extracted from the eye of vehicles, LPS, and BF-5m rats and reverse transcribed into cDNA using Superscript reverse transcriptase system. The expression of caspase 3 and caspase 8 was quantified by qPCR using commercially available rat primers. HPRT was used as internal control. Results are expressed as arbitrary units based on calculation of 2^−ΔΔCt^ method. Relative amount of target genes were normalized to HPRT and to vehicle. Results are expressed as the mean ± SEM of *n* = 6 observations for each group. ^*^ *P* < 0.05 and ^**^ *P* < 0.01 versus LPS-treated rats; °*P* < 0.01 versus vehicle saline group.

Bcl-xl, an antiapoptotic protein, showed slightly appreciable expression in eyes homogenates of EIU rats, while its expression was much stronger in ocular tissue of rats treated with BF-5m (Figure 9). In contrast, mRNAs for caspase 3 and caspase 8 were reduced by BF-5m (Figure 10).

## 4. Discussion

Here we show that the AR inhibitor BF-5m was able to decrease the clinical signs of endotoxic uveitis, an effect that was associated to diminution of the nitrotyrosine and to an increased expression of the antioxidant proteins MnSOD and GPX within the eye. Furthermore, BF-5m decreased the expression of the ubiquitin, 20S and 26S proteasome subunits in uveitic eyes, highlighting that, in addition to stress lowering activities, BF-5m also may lower the deleterious effect that the proinflammatory ubiquitin-proteasome system may have on the integrity of eye structures. As a major system for nonlysosomal intracellular protein degradation in eukaryotic cells, it is involved in a number of biological processes, including inflammation, proliferation, and apoptosis, that are responsible for the disease progression and associate with poor prognosis [19]. The ubiquitin-proteasome system is required for activation of nuclear factor kappa B (NF-*κ*B), a central transcription factor that regulates inflammatory genes, by degradation of its inhibitory kappa B (I*κ*B) proteins [20], and a series of downstream events leading to the development of the inflammatory responses within organs and tissues. Here, the reduction of ubiquitin-proteasome subunits by BF-5m was accompanied by reduced expression of NF-*κ*B in the eyes of EIU rats. Consequently, the levels of proinflammatory factors TNF-*α* and MCP-1 and the proapoptotic [21] caspase 3 and caspase 8 were diminished. Therefore, inhibition of the local AR by BF-5m reduces the stress-induced inflammatory component of the uveitis. The compound also increased the expression of two cell survival and proliferation markers, the CD34 and CD117, within the ocular tissue. CD117 is a transmembrane tyrosine kinase receptor, also called c-KIT, which involves the development and survival of hematopoietic stem cells, melanocytes, germ cells, mast cells, and interstitial cells of Cajal [22]. CD34 is a marker of stem cells; it is a member of a family of single-pass transmembrane proteins that functions as a factor in cell-cell adhesion [23]. Literature data reported that the expression of these markers is stimulated after numerous insults including oxidative stress and inflammation [24, 25] and is aimed at signalizing starting repair of damaged tissue by the EPC recruited to the insulted site. Therefore, although further insights are needed, BF-5m by increasing the recruitment of EPCs into the eye may facilitate the initiation of EIU resolution.

In conclusion, this study shows that the benzofuroxane derivative BF-5m represents a new opportunity for drug intervention aimed at preventing EIU development. It improves the immune-inflammatory profile of the rat eye.
